# Spontaneous Recovery of the Injured Higher Olfactory Center in the Terrestrial Slug *Limax*

**DOI:** 10.1371/journal.pone.0009054

**Published:** 2010-02-08

**Authors:** Ryota Matsuo, Suguru Kobayashi, Jun Murakami, Etsuro Ito

**Affiliations:** Kagawa School of Pharmaceutical Sciences, Tokushima Bunri University, Sanuki, Kagawa, Japan; Centre de Recherches su la Cognition Animale - Centre National de la Recherche Scientifique and Université Paul Sabatier, France

## Abstract

**Background:**

Of all organs and tissues in adult mammals, the brain shows the most limited regeneration and recovery after injury. This is one reason why treating neurological damage such as ischemic injury after stroke presents such a challenge. Here we report a novel mode of regeneration which the slug's cognitive center, the procerebrum, shows after surgical lesioning in the adult. It is well known that the land slug *Limax* possesses the capacity to demonstrate conditioned food aversion. This learning ability critically depends on the procerebrum, which is the higher olfactory center in the brain of the terrestrial mollusk.

**Principal Findings:**

In the present study, after a 1-month recovery period post-surgical lesioning of the procerebrum we investigated whether the brain of the slug shows recovery from damage. We found that learning ability, local field potential oscillation, and the number of cells in the procerebrum (PC) all recovered spontaneously within 1 month of bilateral lesioning of the PC. Moreover, neurogenesis was enhanced in the lesioned PC. However, memory acquired before the surgery could not be retrieved 1 month after surgery although the procerebrum had recovered from injury by this time, consistent with the notion that the procerebrum is the storage site of odor-aversion memory, or deeply involved in the memory recall process.

**Significance:**

Our findings are the first to demonstrate that a brain region responsible for the associative memory of an adult organism can spontaneously reconstitute itself, and can recover its function following injury.

## Introduction

Neurogenesis is possible in some areas of the adult mammalian brain [1]. However, the capacity for neurogenesis is quite limited in mammals, and the spontaneous recoverability of the brain is far from being able to restore the damaged area at the structural level, nor can it recover its normal performance at the functional level.

The central nervous system (CNS) of the adult terrestrial mollusk, on the other hand, has a remarkable regenerative ability [2]. It has been demonstrated that damage to the pedal ganglion of the Pulmonate *Melampus* results in locomotor deficits that recover spontaneously following a recovery period. During this period, reorganization of the neural wirings occurs, and the remaining ganglia compensate for the function of the damaged ganglia by extending new axons [3]. The tentacles of *Helix* and *Achatina* also exhibit regeneration after amputation at both the morphological and functional levels [4], [5]. These data indicate that the CNS of the adult terrestrial mollusk has the capacity to regenerate following injury or ablation. The CNS of animals that are more distantly related to vertebrates, such as *Planaria* and *Enchytraeus*, also have regenerative capacities [6], [7]. These animals, however, have very limited cognitive functions in comparison to terrestrial mollusks, which are capable of higher order forms of learning and memory [8], [9].

Associative memory is one of the most important brain functions. Animals learn the relation between the two events that occur in a temporally contiguous manner, but do not when a certain time interval is given in between [10]. The land slug *Limax* possesses the capacity to demonstrate conditioned food aversion. The pairing of the odor of some foods (e.g. carrot juice) with an aversive stimulus such as the bitter taste of quinidine solution, results in avoidance of that odor thereafter, but does not when the two stimuli were given with an interval of 30 min or 1 hr [8], [9], [11]. This learning ability critically depends on the procerebrum (PC), which is the higher olfactory center located bilaterally in the CNS of the terrestrial mollusk. It consists of ∼10^5^ neurons, and they receive olfactory inputs at three different levels: directly from olfactory sensory neurons, secondary input via tentacle ganglion, and tertiary input via digit (tip of the tentacle) and tentacle ganglion [12]. The PC neurons then give outputs to motor neurons or to other interneurons [13]–[15]. In the PC, the oscillatory frequency of the local field potential (LFP) is modulated in an olfactory input-dependent manner reminiscent of the olfactory bulb of mammals [16]–[20].

We previously reported that the PC is necessary for odor-aversion learning in the slug [21], [22]. Odor-aversion behavior was abolished when conditioning took place 7 days after bilateral surgical lesioning of the PC, while odor-sensing ability remained intact. Post-conditioning lesioning of the PC also interfered with the retention and/or the retrieval of memory. These results clearly demonstrated the necessity of an intact PC for odor-aversion learning and memory.

In the present study, we examined the restoration of the learning ability of the slug 1 month after the PC lesion. We then asked whether the lesioned PC showed recovery at the morphological level and/or at the electrical activity level. Finally, we investigated the recovery of the PC by examining cell proliferation. We also tried to ask whether the PC is the locus of memory storage by taking advantage of the recoverability of the PC.

## Results

### Learning Ability Spontaneously Recovered 1 Month but Not 1 Week after a PC Lesion

**Figure 1:**
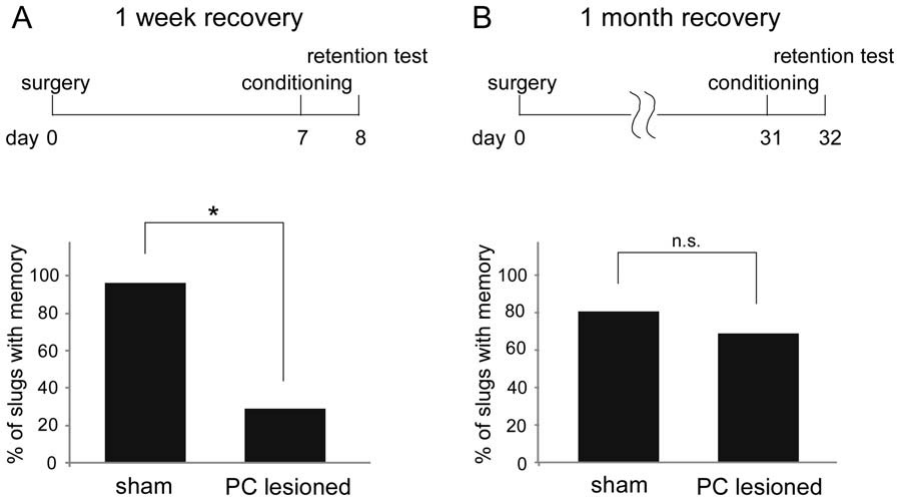
Learning ability recovers 1 month but not 1 week after a PC lesion. (**A**) PC-lesioned slugs were unable to learn following a 1 week recovery period (n = 28 for sham-operated, n = 34 for PC-lesioned slug) with a significantly smaller percentage of lesioned slugs demonstrating odor-aversion conditioned behavior compared to that of the sham operated slugs. *P<0.001 by χ^2^-test. (**B**) PC-lesioned slugs were able to learn following a 1 month recovery period (n = 58 for sham-operated, n = 74 for PC-lesioned slugs) with a percentage of lesioned slugs demonstrating odor-aversion conditioning that was comparable to that of sham operated slugs.

We first examined whether PC-lesioned slugs could demonstrate odor aversion conditioning after a 7 day or a 1 month recovery period. When slugs were conditioned 7 days after sham operation or bilateral PC lesion and then tested following a 24-h retention period, 27 of 28 sham-operated slugs (96.4%) and 10 of 34 PC-lesioned slugs (29.4%) showed avoidance behavior (Fig. 1A; χ^2^ = 28.66, *P*<0.001, d.f.  = 1 in all cases). When slugs were conditioned 1 month after a sham or bilateral PC lesion operation and then tested following a 24-h retention period, 47 of 58 sham-operated slugs (81.0%) and 51 of 74 PC-lesioned slugs (68.9%) exhibited avoidance behavior (Fig. 1B). There was no statistically significant difference between the two groups (χ^2^ = 2.50). These results indicate that the learning ability of the slug was spontaneously restored within a 1 month recovery period.

### Local Field Potential Oscillation Had Recovered 1 Month but Not 1 Week after a PC Lesion

**Figure 2:**
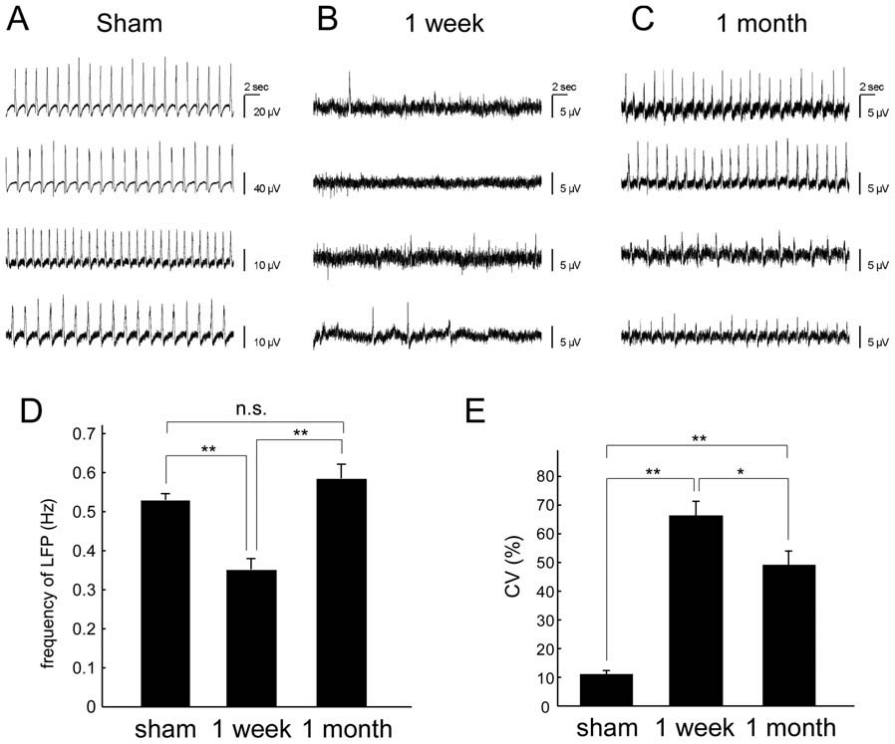
Spontaneous LFP oscillation is restored within a 1 month recovery period. (**A**) Periodic LFP oscillation in the sham-operated PC. (**B**) Infrequent and irregular burstings in the 1 week recovery PC group. (**C**) Periodic LFP oscillation in the 1 month recovery PC group. In (A)–(C), all the recordings were made from different slugs. (**D**) Quantitative data on oscillatory frequency (mean ± SE) show that the frequency was significantly lower 1 week following PC lesion as compared to sham-operated slugs, while 1 month following PC lesion LFP frequency was comparable to that of the sham-operated group. (**E**) Quantitative data on the coefficient of variation (CV) of bursting intervals (mean±SE) in the PC show that the bursting interval 1 week following a lesion was significantly more variable than the sham-operated PC group. While there was some recovery to stable oscillation in the 1 month recovery group, it did not return to a level comparable to that of the sham-operated group. sham, n = 64; 1 week, n = 30; 1 month, n = 64. *P<0.05, **P<0.001 by Student's *t*-test (two-tailed).

Like the olfactory bulb of rodents, the slug PC exhibits LFP oscillation and its frequency is modulated during olfactory information processing [16], [18], [20]. Considering LFP oscillation as an indicator of functional odor information processing, we investigated whether LFP oscillation is restored after a 1 week (i.e. 8 days after the surgery because LFP was recorded just after the memory retention test, see Fig. 1A) or a 1 month recovery period following PC lesioning. To compare LFP oscillations objectively, we focused on the frequency and the periodicity of the LFP bursts (see Materials and Methods for details). Sham-operated groups with 1 week (n = 34) and 1 month (n = 30) recovery periods were grouped together because they did not differ with respect to frequency (*P* = 0.34 by Student's *t*-test) or periodicity (*P* = 0.63 by Student's *t*-test). When bursting frequency was examined following a 1 week recovery period, the LFP (n = 30) of bilaterally lesioned PCs showed infrequent bursting (*P*<0.001 vs. sham, *P*<0.001 vs. 1 month by Student's *t*-test, Fig. 2B, D), whereas slugs allowed a 1 month recovery period after bilateral PC lesioning (n = 64) showed high frequency bursting comparable to that of the sham-operated group (n = 64, Fig. 2A, C, D, *P* = 0.17 by Student's *t*-test). The sham-operated group exhibited a periodic oscillation with constant intervals (Fig. 2A, E), whereas no periodic oscillation was observed in the PC-lesioned group after 1 week of recovery (Fig. 2B, E), with only irregular busting with small amplitudes detected (coefficient of variation (CV) of inter-burst intervals in sham vs. PC-lesioned; *P*<0.001). The periodicity in the oscillatory frequency was substantially restored in the PC-lesioned group after 1 month (*P*<0.05 vs. 1 week, Fig. 2C, E), although irregularity still remained (*P*<0.001 vs. sham). These results suggest that the frequent, periodic oscillation of LFP was restored considerably within the 1 month recovery period.

### Size and Number of Cells Were Restored in the Lesioned PC during a 1 Month Recovery Period

**Figure 3:**
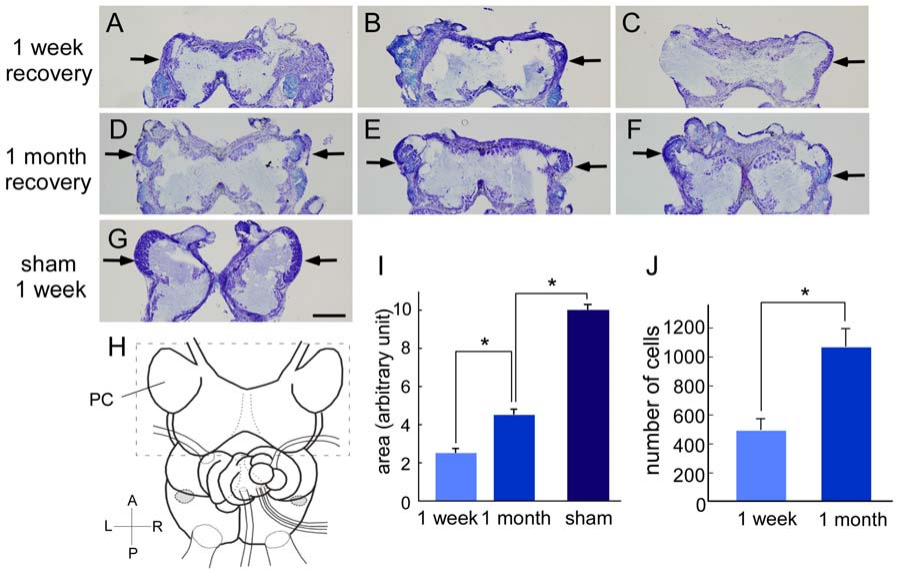
The size and number of cells in the PC is larger in the 1 month recovery group than in the 1 week recovery group. Representative photographs of toluidine blue-stained sections of brains 1 week after a PC lesion (**A–C**), 1 month after a PC lesion (**D–F**), and 1 week after a sham operation (**G**). Only the cerebral ganglia are shown. Arrows indicate the PC. Scale bar, 500 µm. (**H**) A schematic drawing of the slug brain. The rectangle surrounds the cerebral ganglia including the PC. (**I**) Quantification of the sum of the bilateral PC area (1 week recovery, n = 34; 1 month recovery, n = 53; sham, n = 23) showed that the area is significantly diminished one week and one month following lesion, with some recovery in the one month recovery group. (**J**) The number of cells within the PC is larger in the 1 month recovery group (n = 19) than in the 1 week recovery group (n = 18). *P<0.001 by Student's *t*-test. A, anterior; P, posterior; R, right; L, left.

We next investigated the recovery of the PC at the histological level. We measured the size of the PC in toluidine blue-stained sections of the brain after 1 week or 1 month following a PC lesion. Analysis of PC size 1 week (n = 34, Fig. 3A, B, C) and 1 month (n = 53, Fig. 3D, E, F) following lesion showed that the sum of the area of the bilateral PC in the 1 month recovery group was 1.83-fold larger than that of the 1 week recovery group (*P*<0.001, Student's *t*-test, Fig. 3I). When comparing only the larger of the 2 PCs of each slug, the 1 month recovery group had a larger PC area (1.64-fold) than the 1 week recovery group (*P*<0.001, Fig. S1). However, the size of the PC following lesion was not restored to an extent comparable to that of sham-operated slugs (Fig. 3G, H, I). The sham operated group after 1 month of recovery (n = 13) and the sham operated group after 1 week of recovery (n = 10) were grouped together because the size of their bilateral PCs did not differ [mean ± SE: 9.62±0.26 vs. 10.38±0.45 in arbitrary units (au), *P* = 0.14]. The size of the PC in the PC-lesioned 1 month recovery group was 0.45-fold of that of the sham operated group (4.49±0.26 vs. 9.95±0.25 au, *P*<0.001, Fig. 3I), indicating that the size of the PC was not restored completely after 1 month of recovery time.

Next we examined the number of the cells within the PC of randomly chosen animals from the 1 month (n = 18) and 1 week (n = 19) recovery groups. The number of cells in the PC in the 1 month recovery group was, on average, 2.16-fold (*P*<0.001, Student's *t*-test) greater than that of the 1 week recovery group (1074.6±130.0 vs. 497.2±78.3 au, Fig. 3J).

### The Relationship between Memory Performance and PC Size

**Figure 4:**
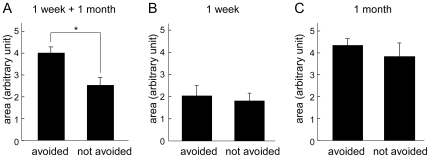
The relationship between the size of the PC and learning ability. (**A**) The size of the PC is larger in the slugs that showed odor-aversion (n = 45) than in the slugs that did not demonstrate aversion when the recovery period was not considered (n = 30). *P<0.005 by Student's *t*-test. (**B**) There is no difference in the size of the PC between slugs that showed odor-aversion (n = 6) and slugs that did not show such aversion (n = 19) in the 1 week recovery group. (**C**) There is no difference in the size of the PC between the slugs that showed odor-aversion (n = 39) and those that did not show such aversion (n = 11) in the 1 month recovery group.

Among 75 slugs that were observed both in the behavioral and in the histological studies, 45 (6 in the 1 week recovery group, and 39 in the 1 month recovery group) displayed conditioned odor aversion to carrot juice, whereas 30 slugs (19 in the 1 week recovery group and 11 in the 1 month recovery group) failed to show such aversion. We found that in these slugs, the sum of the areas of the bilateral PC was significantly larger in the learning-intact (i.e. avoided the carrot juice) groups than in the learning-failed (i.e. touched the carrot juice) groups (4.01±0.26 vs. 2.53±0.34 au, *P*<0.005, Fig. 4A).

We also analyzed the correlation between PC size and behavioral conditioning with respect to recovery time. In the 1 week recovery group, the bilaterally summed PC for the learning-intact slugs (n = 6, 2.04±0.46 au) did not differ from that of the learning-failed slugs (n = 19, 1.82±0.34 au; *P* = 0.74, Fig. 4B). Similarly, in the 1 month recovery group, the bilaterally summed PC for the learning-intact slugs (n = 39, 4.26±0.26 au) also did not differ from that of the learning-failed slugs (n = 11, 3.77±0.58 au; *P* = 0.40, Fig. 4C). These results indicate that successful behavioral conditioning was not correlated with PC size when corrected for recovery period, and that the size of the PC and the number of the neurons in the PC are not the determinants of memory performance. Functional recovery of the PC may also require reorganization of network connectivity.

### Neurogenesis Was Enhanced after PC Lesioning

**Figure 5:**
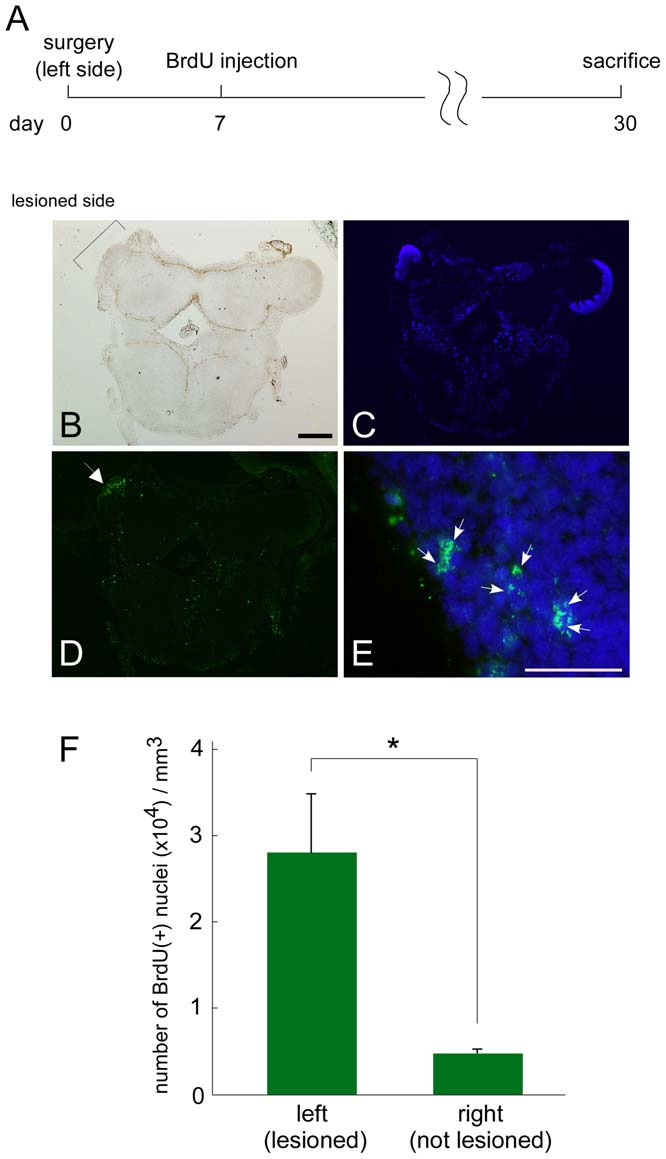
BrdU incorporation is enhanced in the lesioned PC. (**A**) The experimental schedule. (**B**) Bright field microscopic image of the PC. Scale bar: 500 µm. (**C**) Nuclear DAPI staining. (**D**) Immunohistochemical staining of BrdU. An arrow indicates the lesioned (left) PC. (**E**) Magnified image of the left PC. The BrdU signal and the nuclear staining are green and blue, respectively. Arrows indicate pairs of juxtaposed BrdU labeled cells. Scale bar: 50 µm. (**F**) Quantification of the densities of the number of BrdU-positive cells in the lesioned (n = 8) and non-lesioned PC (n = 8). *P<0.005 by Student's *t*-test.

We examined whether the larger number of cells in the PC in the 1 month recovery group compared to the 1 week recovery group (Fig. 3J) was the result of cell proliferation. 5′-bromodeoxyuridine (BrdU) was injected into the body cavity 7 days after the unilateral (left side) PC lesion and the brain was dissected 23 days later (Fig. 5A, B). Using immunohistochemistry, we compared the densities of the cells dually labeled by BrdU (Fig. 5D, E) and DAPI (Fig. 5C, E) between the left (lesioned) and the right (unlesioned) PC. As shown in Fig. 5D & 5F, a substantially larger number of BrdU-labeled cells were found in the lesioned PCs in comparison to the control PCs (2.80×10^4^ vs. 048×10^4^ cells/mm^3^, *P*<0.005, Student's *t*-test). Moreover, the BrdU-immuno-positive signals were juxtaposed to each other in many cases (Fig. 5E), supporting the notion that BrdU-labeling occurred during cell division.

**Figure 6:**
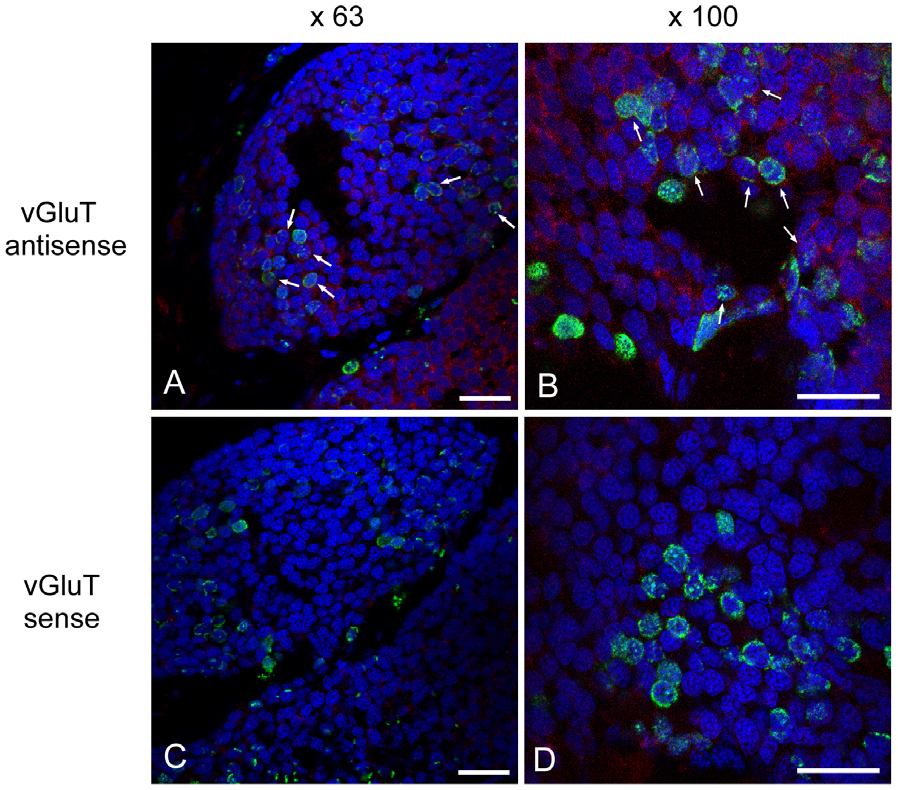
Double staining of BrdU and vGluT mRNA. (**A, B**) Confocal images of sections dually stained by anti-BrdU antibody (green) and an antisense riboprobe against vGluT mRNA (red). Nuclei were stained with DAPI (blue). Arrows indicate BrdU-positive nuclei surrounded by a vGluT signal as demonstrated by *in situ* hybridization. (**C, D**) No signal is detected by a sense probe against vGluT mRNA. Magnifications of objective lenses were 63× (**A, C**) or 100× (**B, D**). Scale bars: 20 µm.

To investigate whether the newly generated cells are neurons or glia, we dually labelled sections of the regenerated PCs with an antibody against BrdU and an antisense riboprobe against vesicular glutamate transporter (vGluT) mRNA. We examined vGluT expression because a majority of neurons in the PC are glutamatergic [23], and vGluT can serve as a molecular marker of glutamatergic neurons [23], [24]. Many of the cells with BrdU-positive nuclei were also stained by vGluT antisense riboprobe, indicating that at least some of the newly generated cells are glutamatergic neurons (Fig. 6A, B). The sense riboprobe did not exhibit any staining signal (Fig. 6C, D). We also examined the expression of *Limax* nitric oxide synthase 2 (limNOS2) gene, which has been recently demonstrated to exist in nonburisting neurons in the PC [25]. Here again, some of the BrdU-positive nuclei were surrounded by the signals of the antisense riboprobe to limNOS2 mRNA (Fig. S2A). The sense riboprobe did not exhibit a signal (Fig. S2B).

### The PC Is the Storage Site of Aversive Olfactory Memory

**Figure 7:**
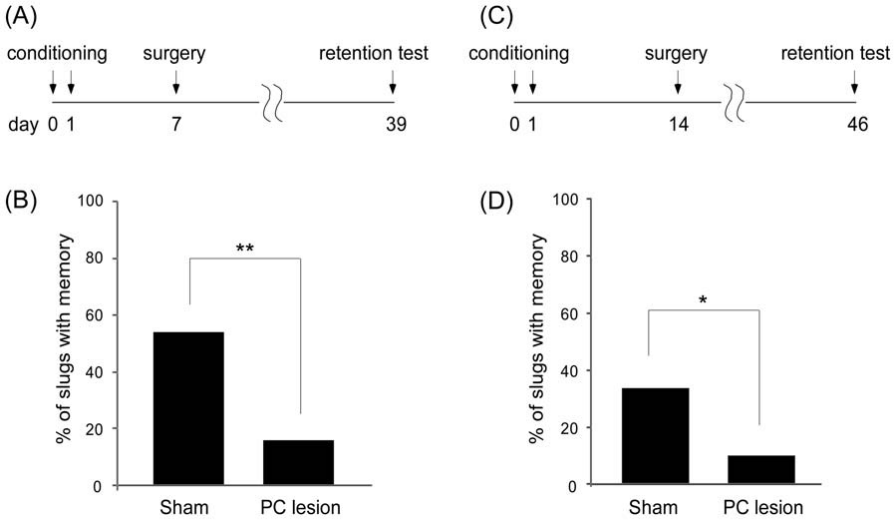
Odor-aversion memory was irreversibly lost after PC lesioning. (**A**) Time schedule of the experiment in which the bilateral PC were lesioned 7 days after conditioning. (**B**) A significantly higher percentage of sham-operated slugs (n = 37) show odor-aversion behavior than do PC-lesioned slugs (n = 38). (**C**) Time schedule of the experiment in which the PC was bilaterally lesioned 14 days after conditioning. (**D**) Sham-operated slugs (n = 30) show better memory retrieval than PC-lesioned slugs (n = 31). *P<0.05, **P<0.001 by χ^2^-test.

In a previous study, we showed that odor-aversion memory could not be retrieved when a bilateral PC lesion was performed 3 h to 7 days following conditioning [21]. However, there remains a possibility that the memory engram still resided in the other parts of the brain, and it could not be retrieved solely because a route for memory retrieval was destroyed by PC lesioning. We thus tried to investigate whether the memory is retrieved after the recovery of the PC. It is known that odor-aversion memory decays within a couple of weeks after a single trial conditioning session [11], [26]. We thus conducted two conditioning trials with a 24-h inter-trial interval to ensure that the memory would persist for more than 1 month (Fig. 7A, see Materials and Methods for details). Seven days following conditioning, the PC was lesioned bilaterally (Fig. 7A). The memory retention test was performed 32 days after the PC lesion. If the PC is the storage site of odor-aversion memory, it is expected that the PC lesion will destroy the memory and retrieval will be absent.

In the sham-operated group, 20 of 37 slugs (55.6%) avoided the carrot juice, whereas a significantly smaller portion, 6 of 38 slugs (15.8%), did so in the PC-lesioned group (Fig. 7B, χ^2^ = 12.12, *P*<0.001 vs. sham-operated group). These data indicate that the acquired memory was still stored in the PC 7 days after conditioning.

In mammals, the hippocampus has been postulated to be the locus where memory is formed and transiently stored. Memory is then transferred to the cortex after a period of time [27]. To test the possibility that the PC in the slug plays a role similar to that of the hippocampus, we also conducted an experiment with a longer interval between conditioning and the PC lesion. In this experiment, the PC was bilaterally lesioned 14 days after conditioning (Fig. 7C). In the sham-operated group, 10 of 30 slugs (33.3%) avoided the carrot juice, whereas only 3 of 31 slugs (9.7%) did so in the PC lesioned group (Fig. 7D, χ^2^ = 5.09, *P*<0.05), indicating that the memory was stored in the PC even at 14 days after conditioning. This finding is consistent with the hypothesis that the PC is the permanent storage site of odor-aversion memory (but see Discussion).

## Discussion

In summary, our results showed that the PC in the slug spontaneously recovers as early as 1 month following lesioning, as measured at the functional, elecrophysiological, and histological levels. Furthermore, newly divided cells expressing a neuronal marker were observed at 1 month following the lesioning, suggesting that PC recovery may involve neurogenesis. Behavioral recovery accompanied reconstitution of the PC, and not merely regeneration of injured nerves. We further found that lesioning as much as 2 weeks after conditioning disrupted avoidance behavior, demonstrating that odor-aversion memory remains dependent upon the PC for at least 2 weeks, a finding that is consistent with the view that the PC is the permanent storage site of odor-aversion memory.

### Spontaneous Recovery of the Adult PC

The requirement of an intact PC for odor-aversion learning has been recently demonstrated by lesion experiments [21], [22]. Our present study demonstrated that the PC has an ability to spontaneously recover and reconstitute itself without any exogenous treatment. This is different from the rodent hippocampal model which requires administration of neurotrophic factors for partial recovery [28], although several studies reported that endogenous neurogenesis and migration of newly generated neurons are enhanced following stroke [29] or focal laser-lesioning [30] in rodents. This also is a novel mode of regeneration, distinct from the previously reported vigorous nerve regeneration in Pulmonates [2], [5]. Our results add a novel example of the robustness of the Pulmonate CNS.

It has long been known that the mammalian cerebral cortex possesses regenerative capabilities when damage occurs during the neonatal period [31], [32]. However, regeneration in the neonate is mediated by morphological changes in the remaining neurons, and not by proliferative neurogenesis. Moreover, this regenerative ability is restricted to the neonatal period.

### Neurogenesis in the PC

It has been proposed by other groups that cellular proliferation continues in the PC of Pulmonates even into adulthood [33], [34]. But it has not clearly been demonstrated whether the newborn cells are neurons or glia. Here we found that many BrdU-positive cells were also positive for vGluT mRNA (Fig. 6) and limNOS2 mRNA (Fig. S2). Because vGluT functions as a filling pump that transports glutamate from the presynaptic cytoplasm into the vesicle lumen [35], it is often considered a molecular marker of glutamatergic neurons in mollusks [23], [24]. limNOS2 has recently been demonstrated to be expressed in nonbursting neurons in the PC [25]. However, due to the scarcity of useful molecular markers in *Limax*, our results do not exclude the possibility that some of the BrdU-positive cells are glial cells, or neurons containing neurotransmitters other than glutamate.

Neuronal progenitor cells reside in the apical area of the PC, and continue to divide and create new neurons throughout the Pulmonate lifespan [33], [34]. In fact, our results demonstrated that there is some proliferative activity in the unlesioned PC (Fig. 5F, right column). These neuronal progenitor cells may be diverted to the source of neurogenic activities during the regeneration of the PC. Quantitative analysis in the present study demonstrated that proliferative activity is enhanced 5.8-fold in response to PC injury (Fig. 5F). Given that BrdU injections took place only on day 7 post-lesion, this value does not necessarily reflect the total proliferative activity. Injection of BrdU at several different time points post-lesioning would reveal the time course of neurogenesis triggered by injury.

We failed to detect any correlation between the size of the recovered PC and the learning ability when corrected for recovery period (Fig. 4B, C). In the case of 1 week recovery group, the result was similar to that in the previous report (Fig. 5 in [21]), where there was no correlation between the remaining size of the lesioned PC and the learning ability in a short recovery period. However, the results do not necessarily discredit our conclusion that the PC is the critical locus for memory formation/storage, because the network rewiring is a distinct aspect from neurogenesis. Moreover, the final size of the recovered PC (1 month-post lesioning) depends on the initial size (and probably the number of the residual progenitor neurons) in the PC just after the surgery.

In very limited cases, we observed no sign of regeneration of the lesioned PC even 1 month after the surgery. This might be caused by a complete removal of all the PC cells, which precluded the regeneration mediated by the remaining neuronal progenitor cells. Future experiments should investigate whether the cells in the outside of the PC have also potential to be diverted to the source of the progenitor cells for the reconstitution of the PC.

### The PC and the Olfactory Bulb

The PC is analogous to the olfactory bulb in mammals in that it receives direct input from the olfactory epithelium and it shows an olfactory-dependent change in the LFP oscillation frequency [16], [17], [19], [36]. However, the PC also receives olfactory information indirectly via the tentacle ganglion in the tips of the superior and inferior tentacles [37], [38]. In addition, some of the afferents from the tentacle ganglion directly enter into the cerebral ganglion, bypassing the PC [37]. The anatomical organization is also different between the olfactory bulb and the PC. Thus far, no glomerulus-like structure has been reported for the PC. The PC, therefore, does not completely correspond to the olfactory bulb of mammals.

The PC and the olfactory bulb do share an important common characteristic in that neurogenesis continues into adulthood [33], [34], [39]–[41]. However, to our knowledge there has been no report that the olfactory bulb has the capacity to restore its size and cell numbers by neurogenesis after bulbectomy in mammals. It would be intriguing to investigate which other distantly related terrestrial animals have the capability of olfactory center reconstitution following injury or ablation.

Wright & Harding (1982) [42] showed that olfactory learning ability spontaneously recovers from bilateral bulbectomy after a lengthy recovery period (>150 days) in mice. However, this functional recovery is not mediated by neurogenesis in the lesioned olfactory bulb but rather by the establishment of new neuronal connections between olfactory sensory neurons and the cortex [43], although the importance of this new connection for olfaction is still controversial [44]–[46]. Although our present results did not decisively demonstrate that the recovery of the inured PC is mediated only by neurogenesis, the ablated PC at least reconstitutes its volume and cell numbers following 1 month. It is possible that the differences in surgical procedures between the previous mouse studies [43], [44] and our study (crushing and squeezing of the cell mass of the PC by fine forceps) may account, in part, for the difference in neuronal regeneration. Complete aspiration in the mouse studies [43], [44] may preclude the neurogenesis potential of the olfactory bulb in mammals. However, this scenario is unlikely. Neuronal progenitor cells reside in the subventricular zone in mice, which is spatially distant from the aspirated olfactory bulbs [41], [47]. In fact, neurogenesis is largely unaffected by bulbectomy in mice [48]. It is more likely, therefore, that the difference in regeneration stems from a difference in the nature of neuronal progenitor cells between slugs and mice. Future screenings are planned to identify the molecules responsible for this difference in the neuronal progenitor cells between these two species.

Wright & Harding (1982) [42] also demonstrated that olfactory memories acquired before bulbectomy can be retrieved after recovery, suggesting that the olfactory memory is not stored in the olfactory bulb. This fact also provides a clear contrast between the PC of slugs and the olfactory bulb of mammals.

### The PC and the Hippocampus

The present studies showed that the PC is not only vital for the formation of odor-aversion learning (Fig. 1A), but also acts as the storage site of odor-aversion memory, or at least is deeply involved in the retrieval of memory (Fig. 7). Odor-aversion behavior was lost after a post-conditioning PC lesion, and was still absent following a full month recovery period. The one month recovery period was sufficient, however, to enable new olfactory associations to take place (Fig. 1B). Irreversible memory loss was evident even when slugs were given a 2-week interval between conditioning and lesioning. These results support the notion that the PC is the storage site of olfactory memory. But there still remains a possibility that the memory stored elsewhere could not be retrieved because the exactly the same neuronal connections within the PC failed to be restored for the retrieval of the memory acquired before the PC lesioning. But our results at least mean that the PC is never a mere simple information conductor because its network reorganization deeply affects the memory recall process, and that some computation is performed in the PC during the memory recall.

Although the mnemonic function of the PC is reminiscent of the hippocampus of mammals, the hippocampus serves as a processing center of configural information as well as a storage site of associative memory. The PC is, therefore, cannot be simply compared to the hippocampus of mammals. Indeed, the role of the hippocampus in memory storage is rather transient, since (after some time) the memory is thought to be transferred to the cerebral cortex where it is stored permanently [27]. The PC contains approximately half of the total number of neurons in the CNS of the Pulmonate [18], and is the convergence locus of conditioned and unconditioned stimuli (i.e. odor information and aversive stimulus) [15], [49]. It is therefore reasonable to theorize that the PC in the Pulmonate acts as a permanent memory storage site. The memory transfer system, as seen in mammals, may have emerged rather recently during evolutionary history, and would have been especially advantageous to higher vertebrates that must process an enormous amount of information in their daily lives.

The LFP oscillation in the PC shows a unidirectional wave propagation [23], [50]–[52], which could potentially be important for separating and integrating neural activity encoding different sensory signals [53]. Recently, the theta oscillations in the hippocampus were also shown to exhibit similar wave propagation [54]. This is another noteworthy example of common characteristics shared between the PC and the hippocampus.

Finally, it is worth noting that the hippocampus and the olfactory bulb are the two areas in the CNS of mammals where the number of neuronal progenitor cells is highest and that neurogenesis continues most remarkably into adulthood [33], [55]. Given the common characteristics such as the mnemonic roles and adult neurogenesis that are shared among the PC, the olfactory bulb, and the hippocampus, an investigation of the evolutionary origin of the PC would be intriguing.

Some of the data have been previously presented in abstract form [56].

## Materials and Methods

### Animals

The terrestrial slugs *Limax valentianus* (adults, 9–16 weeks after hatching) were maintained in our laboratory at 19°C for at least 5 generations as a closed colony. They were fed exclusively on a diet of humidified powder mixture (for its composition, see [19]) and had never eaten carrot before participating in these experiments.

### Surgical Procedure and Behavioral Experiment

Slugs were anesthetized by an injection of ice-cold Mg^2+^ buffer (57.6 mM MgCl_2_, 5.0 mM glucose, 5.0 mM HEPES, pH 7.0) into the body cavity. The surgical lesion of the PC was performed under a stereo microscope. After the PC was exposed bilaterally, the sheath of the PC was pinched and ruptured by sharp forceps. The crushed mass of the PC was squeezed out with the forceps. For the sham operation, the exposed PC was only touched with the tip of the forceps without rupturing the sheath. After surgery, the slug was injected with approximately 200 µl of physiological saline (70 mM NaCl, 2.0 mM KCl, 4.7 mM MgCl_2_, 4.9 mM CaCl_2_, 5.0 mM glucose, 5.0 mM HEPES, pH 7.0) into the body cavity to promote recovery from anesthesia, and was transferred to a plastic container. The slugs were maintained individually, and were fed on the standard humidified powder mixture. Odor aversion conditioning was performed 7 days or 31 days after surgery. Carrot juice served as the conditioned stimulus, and 1% (w/v) quinidine sulfate (Wako Pure Chemicals, Osaka, Japan) solution served as the unconditioned stimulus. Memory retention was tested 24 h after conditioning by an experimenter who was blind to the group designations of the slugs (PC-lesioned group or the sham-operated group). The conditioning was conducted using a shading box, as described elsewhere [57]. In the memory retention test, 1 ml of carrot juice was put in the shape of a half circle with a radius of 90 mm, and the slug was placed just in front of the center of the circle. If it touched the juice within 3 min after passing the center toward the dark side, it was considered to have lost the odor-aversion memory. Otherwise, the slug was considered to retain the memory. For some experiments, animals were subjected to two conditioning trials with an inter-trial interval of 24 h to assure long term memory retention; different conditioning methods were adopted for day 0 and day 1. Day 0 of conditioning was performed conventionally using a shading box. Day 1 conditioning was done in a lighted environment without the shading box and the slugs were surrounded by a carrot juice put in the shape of a half circle with a 45-mm radius, as described previously [57], to prevent escape from the carrot juice. Differences in memory retrieval between the groups were tested for statistical significance using a χ^2^-test.

### Electrophysiology and Data Analysis

Immediately after the behavioral experiments, some slugs (randomly chosen) were deeply anesthetized by an injection of ice-cold Mg^2+^ buffer into the body cavity, and their brains were dissected out. The LFP of the PC was recorded from the surface of the desheathed PC attached to the brain of the slug using a glass suction electrode filled with physiological saline at room temperature. The LFP signal was differentially amplified and bandpass filtered at 0.1–100 Hz (MEG-2100, Nihon Koden, Tokyo, Japan). For recording from the injured PC, we searched for recording positions up to three times by probing the tip of the recording electrode until a field potential could be detected. In the case that only subtle activities could be detected in all of three recordings, we chose the recording data that detected the most prominent burst peaks. For analysis of the burst frequencies and the burst intervals, we extracted the voltage deflection as a peak that had an amplitude larger than the threshold for a duration longer than 10 ms. The threshold was set at the voltage within which 95% of the total recorded points were included throughout the recording period (60 s). The average burst frequency and the CV of the intervals between peaks were calculated for each recording. Both upward and downward voltage deflections were included as peaks, and those with more regular frequencies (i.e. smaller standard deviations of the peak intervals) were selected for data analysis. Group differences in the bursting frequency and periodicity were examined for significance using a Student's *t*-test.

### Histological Analysis of Brain Sections Stained with Toluidine Blue

The slugs were deeply anesthetized by an injection of ice-cold Mg^2+^ buffer into the body cavity, and the whole CNS was dissected out. Some of the brain samples were histologically analyzed after the electrophysiological recordings. The CNS was frozen in Tissue-Tek optimal cutting temperature compound (Sakura, Tokyo, Japan) using liquid nitrogen. Cryostat sections (horizontal, 14 µm-thick) were cut at −16°C in a freezing microtome (Leica, Nussloch, Germany) and every other section was mounted onto a glass slide coated with Vectabond (Vector Laboratories, Burlingame, CA). The sections were dried for 30 min at room temperature, fixed in 10% formaldehyde neutral buffer (Nakarai-Tesque, Kyoto, Japan), and then stained with 0.02% (w/v) toluidine blue solution. After staining, the slides were coverslipped using Permount (Fisher Scientific, MA). Images of the stained sections were obtained using a BX-51 light microscope (Olympus, Tokyo, Japan) with an attached cooled charge-coupled device (CCD) camera DP70 (Olympus) and a 4× objective (NA 0.16). To measure the area of the PC, the experimenter scanned all sections for each subject (usually 20 sections) under the light microscope, and selected sections with: (1) the largest circumference of PC area possible, (2) as thin a cell mass layer as possible to assure selection of sections with equatorial crossings of the PC, and (3) the longest possible arc length of the cell mass layer. Based on these three criteria, the section to be analyzed was chosen from approximately 20 sections, by an experimenter who was blinded with respect to the recovery period of the slug (1 week or 1 month). In the sections, the areas containing densely stained small cell bodies were measured using Canvas X software (Deneba, Victoria, Canada). The area measurement of the residual/reconstituted PC was also performed blindly with respect to the recovery period of the slug. The number of densely stained small cells was also counted within the above mentioned area. Group differences were tested for significance using a Student's *t*-test.

### Immunohistochemistry of BrdU-Labeled Cells

BrdU (0.5 mM) dissolved in physiological saline was injected (0.5 ml/g body weight) into the body cavity twice with a 1-h inter-injection interval, following a 7-day recovery period from a unilateral (left) PC lesion. Brains were dissected out 23 days after the BrdU injections, embedded in Tissue-Tek optimal cutting temperature compound, and rapidly frozen in liquid nitrogen. Sections (12 µm thick) were cut horizontally at −16°C in a freezing microtome (Leica), and the serial sections were alternately mounted onto two different Vectabond-coated slides. One group of slides was used for BrdU immunohistochemistry and the other was used for toluidine blue staining. Following the fixation in ice-cold 70% ethanol/15 mM glycine (pH 2.0) for 20 min, BrdU-immunohistochemistry was performed using a BrdU labeling and detection kit (Roche Diagnostics, Indianapolis, IN) according to the manufacturer's instructions, except that Alexa 488-labeled anti-mouse IgG (Invitrogen, Carlsbad, CA) was used (1∶500 dilution in PBS) as a secondary antibody. The secondary antibody was supplemented with 20 µg/ml RNase A (Nakarai-Tesque) to digest RNAs in the cell body for a 1-h incubation period at room temperature. To visualize the nuclei of the PC cells, all sections were stained with 330 ng/ml (in PBS) propidium iodide (Invitrogen) at room temperature for 5 min, and then coverslipped using Vectashield with DAPI (Vector Laboratories). Images were obtained with a BX-51 light microscope with an attached CCD camera DP70 and objectives (4×, NA 0.16; 20×, NA 0.75). The BrdU/propidium iodide (or DAPI) fluorescent images were superimposed, and the number of dually stained cells was counted. The density of BrdU-labeled nuclei was calculated by dividing the total number of dually stained cells by the volume of the cell mass layer of the PC. The volume of the cell mass layer was calculated based on the photographs of adjoining toluidine blue-stained sections intersecting the PC.

### Double Staining of BrdU and Neuronal Marker mRNAs

Sections (12-µm thick) were prepared as described above, and were fixed in 10% formaldehyde neutral buffer (Nakarai-Tesque) for 30 min. The riboprobe preparation and the conditions for *in situ* hybridization of vGluT and limNOS2 were the same as described previously [16], [18], except that fluorescence signal detection was done using a 2-hydroxy-3-naphtonic acid-2′-phenylanilide phosphate (HNPP) fluorescent detection set (Roche Diagnostics) as substrates for alkaline phosphatase conjugated to anti-digoxigenin antibody (Roche Diagnostics). Following the final wash of anti-digoxigenin antibody, we directly proceeded to immunostaining of BrdU as described above, except that RNase A was not included in the secondary antibody. The sections were coverslipped using Vectashield with DAPI, and imaged using a confocal microscope LMS510 (Zeiss, Göttinggen, Germany) with a 63× objective. The images acquired through different filter sets were superimposed offline by Photoshop CS2 ver. 9.0.2 (Adobe, San Jose, CA). Independent experiments (2 or 3 times) brought about similar, reproducible results.

## Supporting Information

Figure S1 (0.05 MB DOC)

Figure S2 (1.31 MB DOC)
