# Supplementary material for: Spontaneous Recovery of the Injured Higher Olfactory Center in the Terrestrial Slug Limax
Source: PLoS One. 2010 Feb 8;5(2):e9054. doi: 10.1371/journal.pone.0009054 (PMC2816995; doi:10.1371/journal.pone.0009054)
Supplement: Figure S1 — (0.05 MB DOC) [file pone.0009054.s001.doc]

**
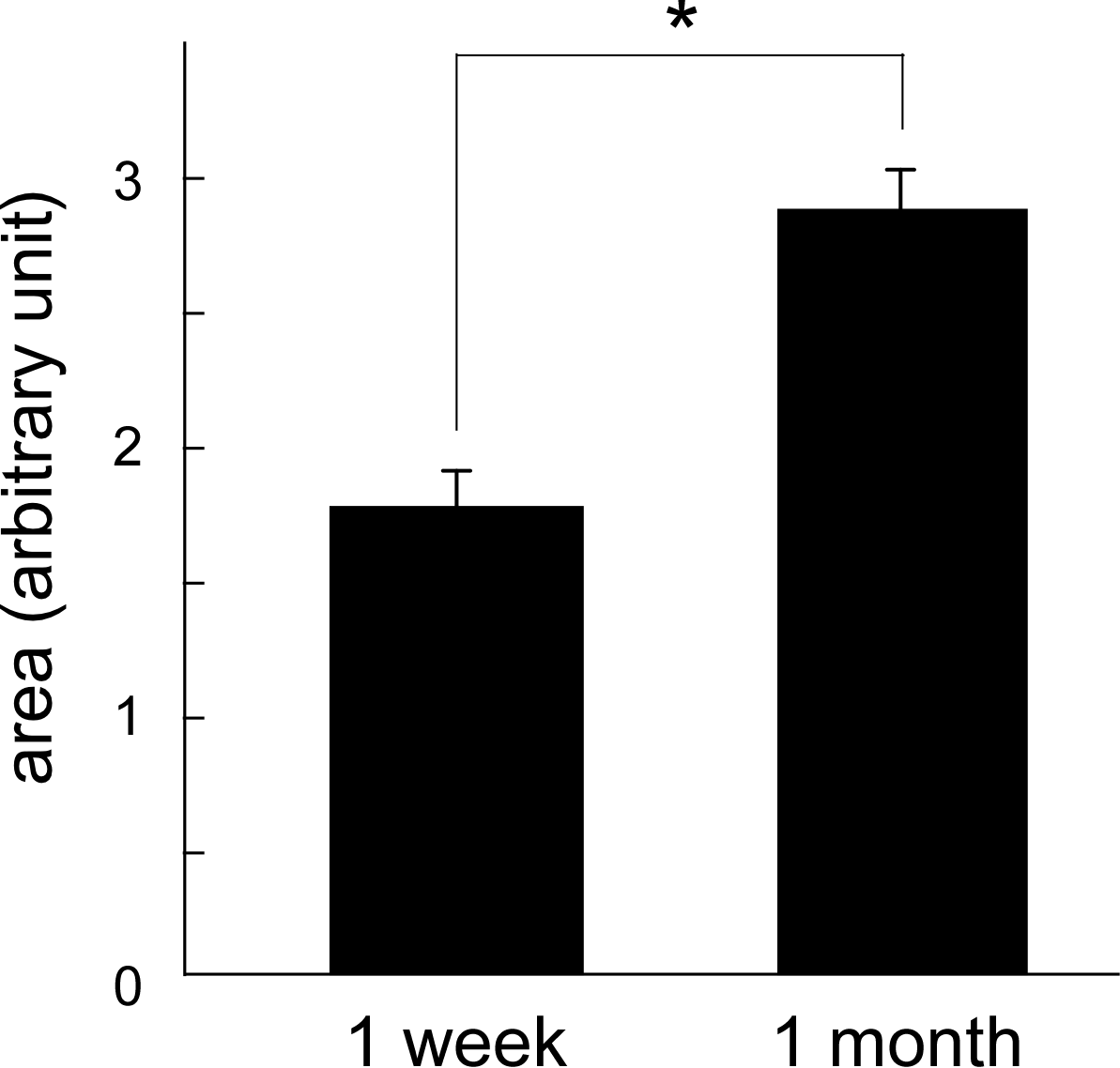
**

**Figure S1.** The larger side of the PC in the 1 month recovery group (n = 53) has a larger area than the larger side of the PC in the 1 week recovery group (n = 34). **P* < 0.001 by Student’s *t*-test.
