# Supplementary material for: Spontaneous Recovery of the Injured Higher Olfactory Center in the Terrestrial Slug Limax
Source: PLoS One. 2010 Feb 8;5(2):e9054. doi: 10.1371/journal.pone.0009054 (PMC2816995; doi:10.1371/journal.pone.0009054)
Supplement: Figure S2 — (1.31 MB DOC) [file pone.0009054.s002.doc]

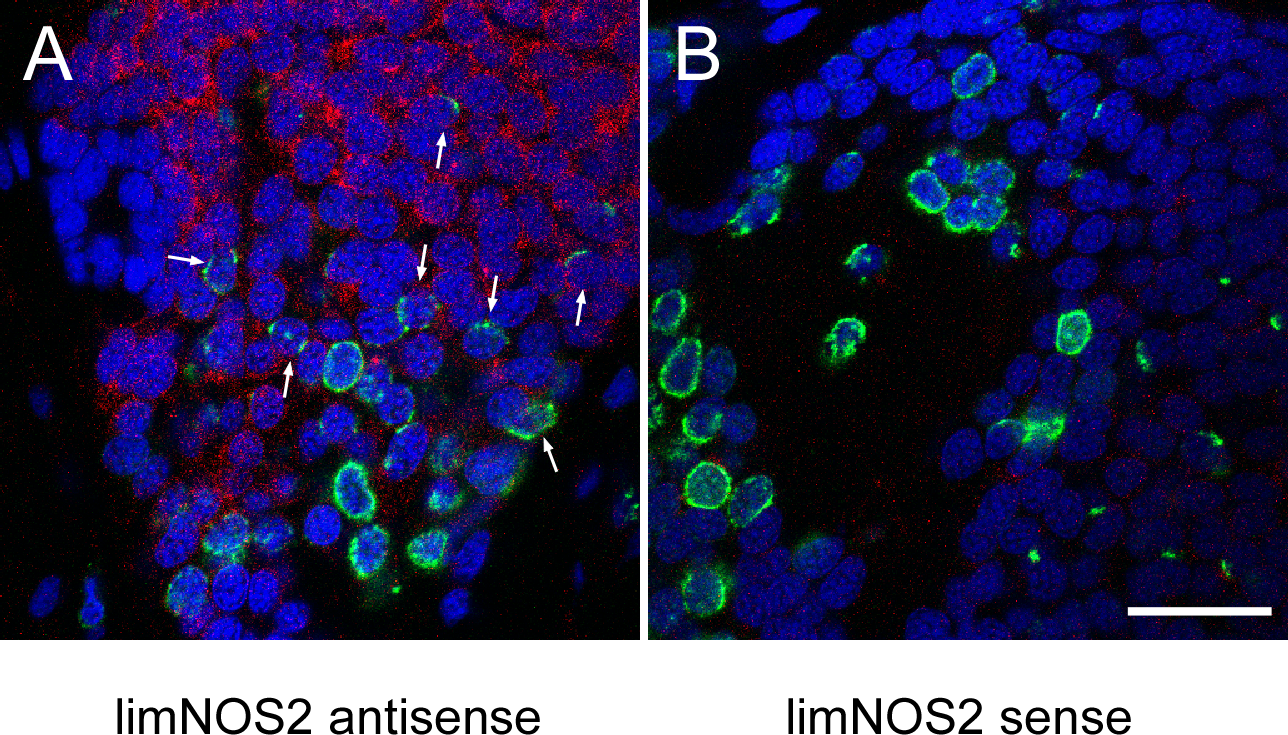


**Figure S2.** Double staining of BrdU and limNOS2 mRNA imaged by confocal microscopy. (A) A confocal image of a section dually stained by anti-BrdU antibody (green) and an antisense riboprobe against limNOS2 mRNA (red). Nuclei were stained with DAPI (blue). Arrows indicate the BrdU-positive nuclei surrounded by positive signals of in situ hybridization to limNOS2. (B) No signal is detected by a sense probe against limNOS2 mRNA. Magnification of objective lens is 100×. Scale bar: 20 μm.
